# Role of anterior piriform cortex in the acquisition of conditioned flavour preference

**DOI:** 10.1038/srep33365

**Published:** 2016-09-14

**Authors:** Cristina Mediavilla, Mar Martin-Signes, Severiano Risco

**Affiliations:** 1 Department of Psychobiology, Cognitive and Behavioural Neuroscience Programme, and Mind, Brain, and Behaviour Research Centre (CIMCYC), University of Granada, Spain; 2 Department of Experimental Psychology and Mind, Brain, and Behaviour Research Centre (CIMCYC), University of Granada, Spain; 3 Department of Pharmacology, and Centro de Investigación Biomédica (CIBM), University of Granada, Spain

## Abstract

Flavour aversion learning (FAL) and conditioned flavour preference (CFP) facilitate animal survival and play a major role in food selection, but the neurobiological mechanisms involved are not completely understood. Neuroanatomical bases of CFP were examined by using Fos immunohistochemistry to record neuronal activity. Rats were trained over eight alternating one-bottle sessions to acquire a CFP induced by pairing a flavour with saccharin (grape was CS+ in Group 1; cherry in Group 2; in Group 3, grape/cherry in half of animals; Group 4, grape/cherry in water). Animals were offered the grape flavour on the day immediately after the training and their brains were processed for c-Fos. Neurons evidencing Fos-like immunoreactivity were counted in the infralimbic cortex, nucleus accumbens core, and anterior piriform cortex (aPC). Analysis showed a significantly larger number of activated cells after learning in the aPC alone, suggesting that the learning process might have produced a change in this cortical region. Ibotenic lesions in the aPC blocked *flavour-taste* preference but did not interrupt *flavour-toxin* FAL by LiCl. These data suggest that aPC cells may be involved in the formation of flavour preferences and that the integrity of this region may be specifically necessary for the acquisition of a CFP.

## Role of anterior piriform cortex in the acquisition of conditioned flavour preference

The goal of food intake is to satisfy metabolic demands, but a large part of the intake in industrialized societies appears to be motivated by the hedonic characteristics of the food. In other words, there is a tendency to eat larger amounts of foods that are especially tasty or sweet. In our daily life, we accept or reject foods according to the hedonic properties that we assign to them after our experience of their intake, and it has been suggested that the prevalence of hedonic over metabolic factors may in part explain the development of obesity and other diseases^1^,^2^. Although some taste preferences and aversions appear to be governed by non-learned mechanisms based on innate predispositions, the development of preferences and aversions for given foods often derives from experience. Thus, the reward value of foods is modified by the association of flavours with their post-ingestive (oral and visceral) consequences, leading animals to develop the corresponding preference or aversion. Flavour aversion learning (FAL) and conditioned flavour preference (CFP) facilitate animal survival and play a major role in food selection, but the neurobiological mechanisms involved are not completely known. The labelling of immediate early genes, such as c-*fos*, has been widely utilized to define the brain areas involved in FAL^3^,^4^,^5^.Thus, the first objective of this study was to examine the neuroanatomical bases of conditioned flavour preference (CFP) by using Fos immunohistochemistry to record neuronal activity and obtain a cell correlate of CFP similar to that identified for FAL^3^,^4^. Flavour contributes to the hedonic evaluation of food, and flavour-taste learning appears to play a critical role in modelling human dietary preferences^6^,^7^. In the present study, we promoted acquisition of a *flavour-taste* CFP by associating a neutral flavour with the sweet taste of saccharin. Because saccharin has no nutritious properties, flavour-taste preference would be exclusively based on hedonic taste and olfactory mechanisms. After induction of this learning, the brains of the animals were processed for the detection of c-Fos protein in order to identify active brain areas after acquisition of a flavour preference. The infralimbic cortex (IL) and nucleus accumbens (NAc) were selected for examination because they have both been related to CFP^8^,^9^,^10^, and in the piriform cortex (PC). This has traditionally been considered as the primary olfactory cortex and was recently proposed to function as an olfactory association cortex similar to the association cortex in other sensorial systems^11^. Thus, this region has been found to contain neurons in which olfactory and taste information converges, permitting flavour perception^12^,^13^. Moreover, it maintains anatomic connections not only with the olfactory bulb but also with associative regions such as the orbitofrontal cortex, IL cortex, hippocampal formation and amygdala, among others^11^,^14^. These anatomic connections would allow participation of the piriform cortex in learning based on olfactory cues^15^,^16^. The data obtained in this immunohistochemical study appear to indicate a role for the aPC in flavour-taste learning, leading us to investigate whether lesions in this region would also affect the acquisition of a CFP. In addition, we decided to study the role of the aPC in FAL, because it has been found to contain more neurons that respond to olfactory cues predicting positive (sucrose) outcomes in comparison to those that respond to olfactory cues predicting negative (quinine) outcomes^15^. Accordingly, we performed experiments to determine the effect of bilateral ibotenic lesions in the aPC on *flavour-taste* CFP induced by saccharin and on *flavour-toxin* FAL induced by LiCl.

## Results

### Experiment 1: Expression of c-Fos in the anterior piriform cortex after acquisition of conditioned flavour preference

Three groups (group 1, 2, and 3) of rats underwent eight alternating one-bottle sessions to acquire a *flavour-taste* CFP. In a fourth control group (group 4), the same flavours were presented in saccharin-free water. On the day immediately after the acquisition sessions, animals in groups 1, 2, and 4 were offered the grape flavour (7 ml) in an attempt to detect anatomical differences in genetic activity according to the previous association or not of this flavour with saccharin. Thus, each animal in these groups consumed 7 ml of grape, corresponding to the minimum volume ingested by an animal in the last two days of acquisition sessions. This procedure was designed to avoid that differences in cell activity could be related to differences in the amount consumed prior to sacrifice. Two hours after this intake, these rats were deeply anesthetized and their brains were extracted, and immunohistochemically processed for c-Fos. Animals in group 3 underwent a two-bottle preference tests (CS+ *vs* CS−).

#### Acquisition of CFP

**Figure 1:**
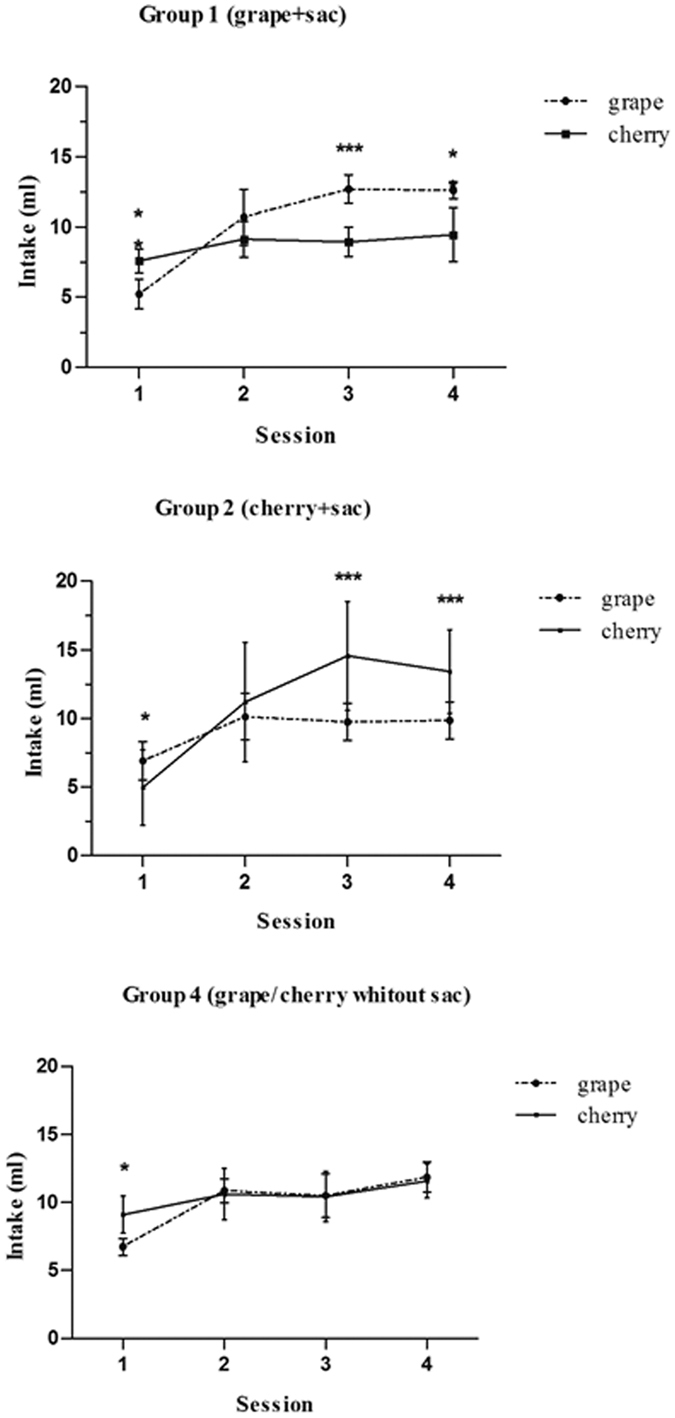
Intake (mean ± SEM) of CS+ and CS− flavours across acquisition sessions in the control group (group 4) and experimental groups (1 and 2). Rats in groups 1 and 2 increased their intake of CS+ over the course of the sessions, whereas those in group 4 consumed similar amounts of each stimulus during this period, showing no preference for one flavour over the other. Saccharin was associated with the grape flavour in group 1 (**a**) and with the cherry flavour in group 2 (**b**). Both flavours were diluted in water in group 4 (**c)**. *p < 0.05; **p < 0.01; ***p < 0.001.

Figure 1 depicts the intake (mean ± SEM) of CS+ and CS− flavours by groups 1, 2, and 4 across the acquisition sessions. The repeated-measure ANOVA (group × trials × fluid) (3 × 4 × 2) revealed that the trials variable (*F*_3,36_ = 107.52, *P* < 0.001), group × trials interaction (*F*_6,36_ = 4.30, *P* < 0.005), group × fluid interaction (*F*_2,12_ = 4.04, *P* < 0.05) and group × trials × fluid interaction (*F*_6,36_ = 17.89. *P* < 0.001) were significant. Newman-Keuls *post-hoc* tests revealed significant differences (*P* < 0.05) between grape and cherry intake in groups 1 and 2 during all sessions except for the second (significant results are indicated in the figure legend). According to these findings, rats in groups 1 and 2 increased their CS+ intake over the course of the sessions, regardless of the flavour (grape or cherry) associated with saccharin. Animals in group 4, as expected, consumed similar amounts of each stimulus during this period, showing no preference for one flavour over the other except in the first trial (*P* < 0.05).

#### Effects of Conditioned Flavour Preference on Fos expression

**Figure 2:**
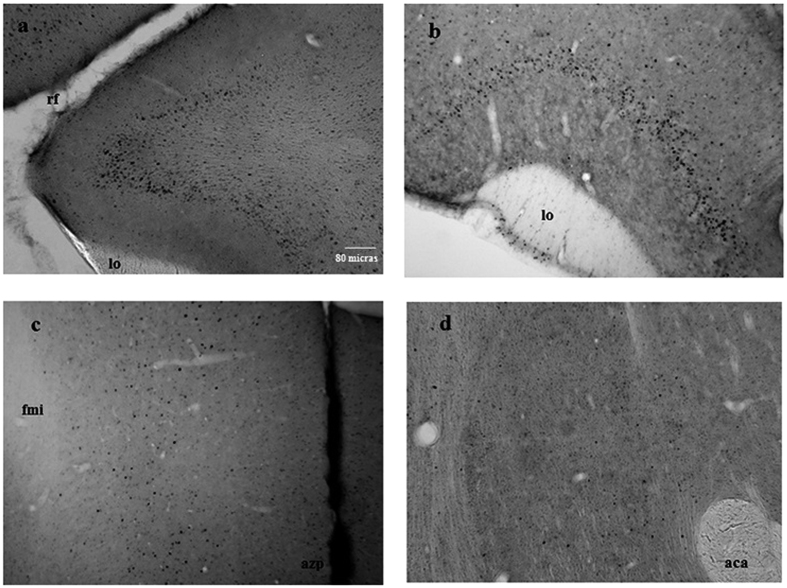
Representative photomicrographs (10x) of the studied regions. (**a**) rostral aPC; (**b**) caudal aPC; (**c**) IL; (**d**) NAcC. rf, rhinal fissure; lo, lateral olfactory tract; fmi, forceps minor of the corpus callosum; azp, azygos pericallosal artery; LV, lateral ventricle; aca, anterior commissure, anterior part.

**Figure 3:**
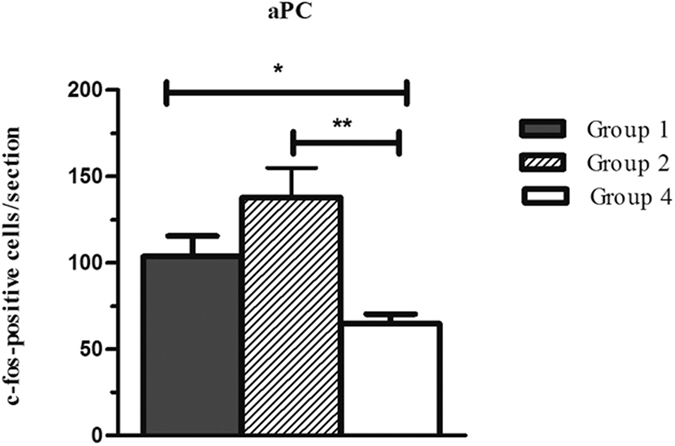
Expression of c-Fos in the anterior piriform cortex (aPC) under the different experimental conditions. Groups 1 and 2 differed in the n° of c-Fos-positive cells from the control group (group 4) but not from each other Data are expressed as mean number ± SEM of c-Fos-positive cells *p < 0.05; **p < 0.01.

**Figure 4:**
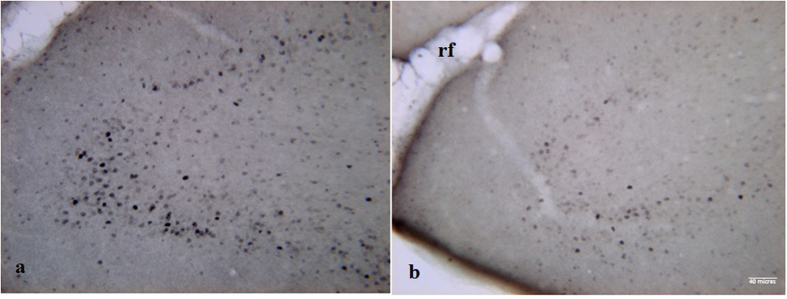
Representative photomicrographs (20x) depicting c-Fos expression in the anterior piriform cortex (aPC) of animals in Group 1 (**a**), and Group 4 (**b**).

Neurons showing Fos-like immunoreactivity were counted in the IL, nucleus Accumbens core (NAcC), and anterior piriform cortex (aPC) of groups 1, 2, and 4 (Fig. 2). The number of c-Fos-positive cells in each region was analyzed by one-way ANOVA, which showed that the aPC was the only region with significant differences among the groups (F_2,12_ = 8.652, *P* < 0.001). The Newman-Keuls *post-hoc* test revealed that groups 1 (*P* < 0.05) and 2 (*P* < 0.01) differed in the n° of c-Fos-positive cells from the control group (group 4) but not from each other (Figs 3 and 4).

#### Two-bottle Preference Test

**Figure 5:**
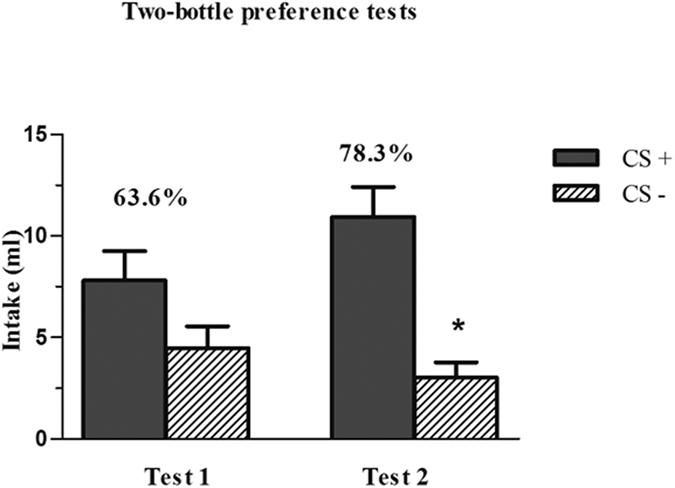
CS+ and CS− intake of Group 3 in the Test 1 and Test 2 preference tests of Experiment 1. Behavioural data showed the animals in this group to be capable of learning the flavour-saccharin association under these experimental conditions. Numbers above bars indicate mean percentage intakes of the CS+. Data are means ± SEM. *p < 0.05.

One-way ANOVA (CS+ *vs*. CS−) analysis of the data for group 3 (Fig. 5) in Test 1 showed that, although the intake of CS+ was higher, the difference between the flavours did not reach statistical significance (*F*_1,5_ = 1.92, *NS*). The difference in intake between CS+ and CS− was significant in Test 2 (*F*_1,15_ = 14.99, *P* < 0.05), in which all animals preferred CS+. Test 2 highlights that the flavour-saccharin association was retained, despite the time interval (6 days) since the acquisition sessions. The behavioural data showed the animals in this group to be capable of learning the flavour-saccharin association under these experimental conditions.

### Experiment 2: Effects of ibotenic lesions in the aPC on *flavour-taste* CFP and FAL induced by LiCl

The data obtained in Experiment 1 appear to indicate a role for the aPC in flavour-taste learning. We performed experiment 2 to determine the effect of bilateral ibotenic lesions in the aPC on *flavour-taste* CFP induced by saccharin and on *flavour-toxin* FAL because it has been found to contain more neurons that respond to olfactory cues predicting positive (sucrose) outcomes in comparison to those that respond to olfactory cues predicting negative (quinine) outcomes^15^.

#### Acquisition of flavour-taste preference

The intake (mean ± SEM) of both CS+ and CS− flavours across acquisition sessions in the sham-lesioned control group and the group lesioned with bilateral infusion of ibotenic acid was analyzed using repeated-measures ANOVA (group × trials × fluid) (2 × 4 × 2), which showed that the group variable (*F*_2,24_ = 6.23, *P* < 0.01) and trials x fluid interaction (*F*_2,48_ = 40.82, *P* < 0.01) were significant. These analyses indicate that all rats increased their intake of CS+ over the course of the sessions. Further analysis showed that the intake of the flavour associated with water (CS−) remained stable, but the intake of CS+ was significantly higher in the final *versus* first session in all groups (*P* < 0.01).

#### Two-bottle Preference Tests

**Figure 6:**
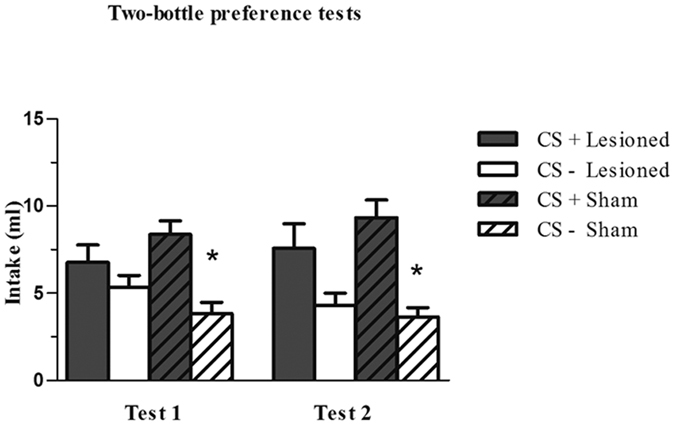
Effects of bilateral ibotenic lesions in the aPC in the Test 1 and Test 2 preference tests in the sham-lesioned control group and lesioned group of Experiment 2. Data are means ± SEM. *p < 0.05.

Figure 6 shows the CS+ and CS− intake of the control and lesioned groups in the Test 1 and Test 2 preference tests. Three-way repeated-measures ANOVA (group × test × fluid) (2 × 2 × 2) revealed a significant effect of the fluid variable (*F*_1,13_ = 47.71, *P* < 0.001) and group x fluid interaction (*F*_1,13_ = 6.51, *P* < 0.05). Newman-Keuls *post hoc* tests showed a significant difference between the groups in their consumption of CS+ (*P* < 0.05). Separate one-way repeated-measures ANOVA revealed that the differences between CS+ and CS− intake were only significant in the sham-lesioned group in Test 1 (*F*_1,7_ = 10.84, *P* < 0.05) and Test 2 (*F*_1,7_ = 16.43, *P* < 0.01).

#### Body weight

Repeated-measure ANOVA (group x day) was used to analyze animal weight during the experimental sessions and showed significance for the main effect of days (*F*_8,104_ = 181,34, *P* < 0.001), with both groups showing the same increase in body weight over the days.

#### Flavour aversion learning

**Figure 7:**
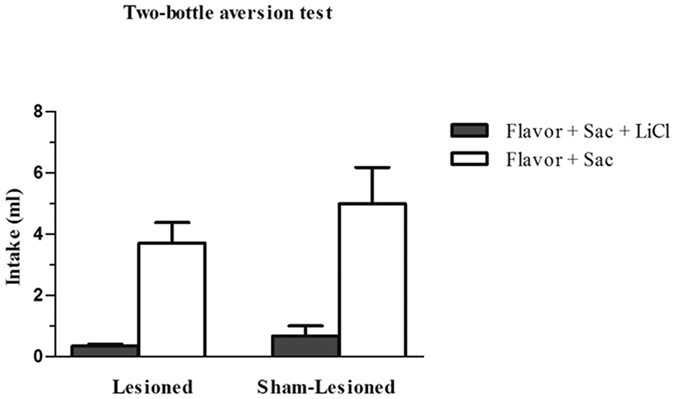
CS+ and CS− intake on the choice aversion test on FAL in the sham-lesioned group and lesioned group with bilateral infusion of ibotenic acid in the aPC. Data are means ± SEM. *p < 0.05; **p < 0.01.

Effects of bilateral lesions in the aPC on the two-bottle aversion test are depicted in Fig. 7. A two-way repeated-measures ANOVA (group × fluid) revealed a significant effect of the fluid variable (*F*_1,13_ = 24.81, *P* < 0.001) but not the group variable or group × fluid interaction. These data indicate that the lesioned and control groups both learned to avoid the flavour associated with LiCl administration. Separate one-way repeated-measures ANOVA showed that the differences between CS+ and CS− intake were significant in both groups (lesioned group: *F*_1,6_ = 26.53, *P* < 0.01; control group: *F*_1,7_ = 10.73, *P* < 0.05).

## Discussion

The induction of a flavour-taste preference increases the number of activated cells in the aPC. To our best knowledge, this represents the first report of a relationship between the aPC and CFP. Immunohistochemical analysis revealed significant differences in the number of c-Fos-labelled cells in the aPC as a function of the procedure applied during the acquisition sessions. Under these conditions, an increase in cellular activity in the aPC was observed in the animals, indicating that an initially neutral flavour had acquired a motivational significance that could be used to manifest preferences (groups 1 and 2). This increase was not observed in group 4, in which the two flavours were diluted in water and were therefore equally preferred across the different sessions; hence, the experimental conditions of this group did not permit flavour-taste learning, in which the positive hedonic value of a flavour would be increased. This result also rules out the possibility that the increased cell activity observed in groups 1 and 2 was merely related to the sensorial processing of the flavours.

c-Fos-positive cells were also detected in the IL and NAcC, but their number did not significantly differ among the groups, despite the reported relationship of these regions with flavour preference acquisition^8^,^9^,^10^. In fact, the medial prefrontal cortex contains neurons that are at least equally effective as gustatory cortex cells to detect palatability, likely attributable to the information received from the gustatory cortex and basolateral amygdala^17^,^18^. Likewise, the NAcC has also been found to contain cells that encode the palatability of sucrose reinforcers^19^. On the other hand, some researchers observed that neurotoxic lesions in the medial prefrontal cortex of monkeys had no effect on the expression or acquisition of taste learning^20^, and an immunohistochemical study found no relationship between c-Fos expression in the IL and an olfactory learning task^21^. Alternatively, it is possible that both regions participate in processing the rewarding aspects of a given flavour in a similar manner to the amygdala, encoding hedonic aspects of a taste but not being involved in the associative significance of taste cues that permits taste learning^20^,^22^,^23^. In the present study, given that groups 1, 2, and 4 drank grape diluted in saccharin-free water in the final session, it appears reasonable to suppose that the IL and NAcC are equally active in all of the animals. For a water-deprived animal, the mere possibility of drinking can itself be rewarding, and the activity observed in these regions may correspond to this aspect of intake, given the role of the medial prefrontal cortex and NAcC in reward^24^,^25^.

The behavioural results obtained in group 3 confirm that these experimental conditions are adequate to promote the acquisition of a preference, with a neutral flavour acquiring motivational significance through its association with an innately preferred taste such as sweetness^26^,^27^. The consumption by this group of a larger amount of CS+ in both choice tests indicates that the animals learned the association. The fact that the consumption of CS+ and CS− did not significantly differ in Test 1 is likely attributable to the experimental conditions required for the immunohistochemical study. Thus, in order to avoid exposure of groups 1, 2, and 4 to the olfactory component of the cherry flavour, the choice test for group 3 was performed after the sacrifice of the animals in the other groups, i.e., some hours after the usual intake time. The results of Test 2, conducted five days later, in which 100% of animals consumed significantly greater amounts of CS+ *versus* CS− indicate that the flavour-saccharin association was established during the acquisition sessions. These findings also demonstrate that the CFP is consistent and long-lasting, persisting for at least five days after an extinction session (Test 1).

Bilateral neurotoxic lesions of the aPC blocked the acquisition of a *flavour-taste* CFP in two-bottle preference tests, whereas the control group learned the association between a flavour and saccharin, consuming significantly more CS+ than CS− in the choice preference test. The capacity to differentiate between sweetened and unsweetened solutions did not appear to be affected by the ibotenic lesions, given that both lesioned and sham-lesioned groups increased the intake of the saccharin-associated flavour across acquisition sessions.

These findings confirm once more that the proposed conditions are appropriate for CFP acquisition in both sham- lesioned and intact animals (Group 3 in Experiment 1). The lesions also appear to affect the capacity to acquire a *flavour-taste* preference, but they did not impede FAL induced by a single administration of LiCl. Alongside the finding that the lesions had no effect on the body weight of the animals, these results allow us to rule out the possibility that the data obtained in CFP were attributable to any generalized or sensory (flavour) incapacity.

The piriform cortex would integrate olfactory information from the olfactory bulb with input from higher-order associative regions, such as the prefrontal cortex, amygdala cortex, and hippocampal formation^11^. Indeed, the piriform is not only related to olfactory coding but may also participate in olfactory memory, given that it is reciprocally connected with associative regions such as the orbitofrontal cortex, IL cortex, hippocampal formation, and amygdala, among others^11^. Thus, it has been postulated that the aPC mediates in the learning and memory of olfactory associations rather than representing purely olfactory information^11^, and it has been related to different associative learnings involving olfactory cues^15^,^16^. Hence, aPC cells demonstrate the plasticity required to participate in different learnings^16^,^28^ (present data). Specifically, an increased connectivity has been observed in pyramidal cells of the aPC after olfactory learning, and it has been suggested that descending OFC-aPC connections modulate aPC cells in order to respond to a particular odour in an appropriate behavioural context, such that these connections are reinforced after the learning^28^. The activity of piriform cortex cells has also been reported to alter when the reward value of the odour is modified^29^. In the present study, if the piriform exclusively processed olfactory signals, we would have found the same number of c-Fos-active cells in all animals, but an increase was only observed in those animals for which the hedonic value of the flavour was modified after its association with a motivationally significant stimulus.

It is also appears relevant to investigate the role of the piriform cortex in FAL because it is known to process more positive than negative olfactory cues, as noted above^18^. In the present study, the aPC lesions did not interrupt FAL induced by LiCl in a two-bottle aversion test, indicating that this cortical region may be relevant in learning that has positive but not negative postingestive consequences. Alternatively, the aPC may play a role in flavour-taste learning based on the hedonic signals of the stimuli rather than on orosensorial-viscerosensory associations, in which the postingestive consequences determine the learning of a preference for or aversion to a food. This proposition is supported by the present study, in which the aPC appear to intervene in the association of a flavour with an orosensory stimulus (sweet taste) but to be unnecessary when the association is established with a visceral signal (LiCl). However, given that structures such as the amygdala, parabrachial complex, and lateral hypothalamus have been related to both preferences and aversions in *flavour-taste* and/or *flavour-nutrient* learnings^30^, further studies using different orosensory (e.g., quinine) and visceral (e.g., intragastric nutrients) stimuli are warranted to determine the precise role of the aPC in the acquisition of flavour preferences and aversions.

The piriform cortex has two subdivisions (anterior and posterior) that differ physiologically and functionally^11^,^15^. Only the aPC was examined in the present study, and it is also necessary to investigate the posterior piriform cortex (PPC). The PPC has been found to contain not only neurons that respond to odours or purely gustatory information alone but also individual cells that respond to both taste and olfactory stimuli^12^, a convergence that enables flavour learning^13^,^31^. In this context, electrophysiological studies have detected changes in the activity of PPC neurons in odour reversal learning tasks, suggesting that representations in the PPC are highly associative^13^. Investigation of the PPC may also help explain why no increase in c-Fos expression was observed in the piriform cortex after acquisition of an olfactory learning task in a study that examined the entire PC^19^. The present study examined flavour learning rather than olfactory learning, based on stimuli whose perception results from the integration of sensations such as taste and retronasal olfaction^7^,^32^,^33^. Hence, the piriform cortex may be part of a “flavour network”, given that the convergence between taste and olfactory signals that allow flavour perception and learning is produced in the piriform cortex even earlier than in the orbitofrontal cortex^12^.

Further studies are required to determine the functional relevance of the piriform cortex in taste learning. In the meantime, the present data appear to indicate that the aPC may function not as a primary olfactory region but rather as an association cortex necessary for learnings such as flavour-taste learning, in which the positive hedonic value of the flavour is increased.

## Methods

### Animals

Male Wistar rats weighing 240–300 g were provided by Janvier (France). They were randomly distributed into experimental groups and individually housed in methacrylate cages that served as training chambers during the experiment. The room was maintained at a constant temperature of 21°C under a 12:12 light-dark cycle, with lights on at 07:30. Food and water were available *ad libitum* except when otherwise reported. All procedures were carried out in accordance with guidelines established by the European Union (2010/63/EU) and Spanish Royal Law 23/2013 and were approved by the Ethical Committee for Animal Research at the University of Granada.

### Experiment 1. Expression of c-Fos in the aPC after acquisition of CFP

Animals were placed on restricted water access and trained to drink tap water for 15 min a day. Water consumption, food intake, and bodyweight were measured daily, and there was a period of re-hydration in the afternoon. After four days of training sessions, three groups (group 1, 2, and 3) of rats underwent eight alternating one-bottle sessions to acquire a *flavour-taste* CFP. The CS+ was an 0.15% saccharin solution flavoured with 0.05% (w/w) non-sweet cherry or grape flavour (Kool-Aid, General Foods, White Plains, NY), and the CS− was the same flavour diluted with tap water. In a fourth control group (group 4), the same flavours were presented in saccharin-free water. Assignation of the CS+ in the groups was as follows: Group 1 (n = 5), grape; Group 2 (n = 5), cherry; Group 3 (n = 6), grape in half of animals and cherry in the other half; Group 4 (n = 5): grape/cherry diluted in water.

After successive acquisition trials, animals in groups 1, 2, and 4 were offered the grape flavour (7 ml) during a 15-min session. Two hours later, these rats were deeply anesthetized (80 mg/kg), and transcardially perfused with phosphate-buffered saline (PBS) and 4% paraformaldehyde. Their brains were extracted, post-fixed in paraformaldehyde overnight and soaked in sucrose (30%) for cryoprotection, and immunohistochemically processed for c-Fos. Animals in group 3 underwent a choice-test (Test 1) on day 13, in which two tubes containing the flavours used as CS+ and CS− were simultaneously presented. Five days later, they underwent another choice test (Test 2) in which the animals had access to the same flavours but in inverse positions with respect to their placement in Test 1.

#### c-Fos Immunohistochemistry

A block of rostral tissue up to the optic chiasm containing the regions of interest (IL, NAcC, and aPC) was sectioned with a cryostat into 50-μm thick coronal slices, which were processed for Fos-immunostaining as previously described^34^,^35^. Slices were rinsed (3x PBS), incubated for 20 min in 0.3% H_2_O_2_ in absolute methanol to quench endogenous tissue peroxidase, rinsed (3x, PBS), and incubated for 1 hr in 1% gelatin-3% normal goat serum in PBS. Slices were transferred to the primary antibody solution of 0.005 g/ml polyclonal rabbit antiserum (mixed in PBS) (Santa Cruz Biotechnology, Santa Cruz, CA; 1:20,000). After 48 hr of incubation at 4°C, slices were rinsed (10x, PBS, 2 hr) and processed by using the ABC method (Vector Laboratories, Burlingame, CA), and the diaminobenzidine substrate was intensified with nickel sulphate to visualize the presence of cell nuclei with c-Fos-like immunoreactivity. Finally, slices were rinsed in PBS, mounted on slides, and coverslipped with DPX.

#### Scoring Immunohistochemistry

Immunoreactivity to Fos was evaluated by examining the preparations under a light microscope and capturing the images with a camera linked to a computerized image-analysis system in a personal computer. For the quantitative analysis, representative sections of the IL and NAcC were selected in each animal; in the case of the aPC, two sections separated by 1.5 mm were selected due to its large size, and the counts were averaged into a single score for each animal. The count was done by a researcher (BJC) blinded to the experimental conditions of the study using ImageJ image analysis software.

### Statistical analyses

Values are expressed as means ± SEM, and statistical analyses were performed using SPSS Statistics 20. A three-way repeated-measures ANOVA (group × trials × fluid) was used to analyze the mean intakes of flavours during the four acquisition trials in Groups 1, 2, and 4. Mean intakes during the preference tests (CS+ *vs*. CS−) in Group 3 were analyzed by one-way repeated-measures ANOVA. The CS+ preference index in the choice tests was also calculated as the percentage of CS+ intake (100x CS+ intake/total intake). For the quantitative analysis of c-Fos immunoreactivity, the mean number of positive c-Fos cells per region was evaluated by one-factor (brain site) ANOVAs for inter-group comparisons. When a significant *F* was obtained, Newman-Keuls *post-hoc* tests were performed to assess specific comparisons. Statistical significance was established at the 5% level.

### Experiment 2: Flavour-taste preference and flavour-toxin aversion learning after ibotenic lesions in the aPC Surgery

Rats were deeply anesthetized with a mixture of ketamine (86 mg/kg) and xylazine (12.9 mg/kg) and positioned in a stereotaxic device. The rats in the lesioned group received an injection of ibotenic acid (10 mg/ml in PBS, Sigma, Madrid, Spain) at anatomical coordinates obtained from the rat brain atlas of Paxinos and Watson^36^: 2.76 mm anterior to the bregma, 4.0 mm lateral to the midline, and 7 mm below the skull surface. The rats in the sham group received an injection of the vehicle only (PBS) and the tip of injection needle was placed at 1 mm above the lesion coordinates. Ibotenic acid and vehicle were administered through a 5.0 μl Hamilton micro-syringe driven by an infusion pump (KD Scientific Inc., MA, USA). Infusions were delivered in an injection volume of 0.4 μl/side at a flow rate of 0.05 μl/min. After each infusion, the needle remained in place for 2 min to allow diffusion of the solution into the tissue. During the recovery period of intervention, one animal died in the lesioned group, which finally comprised 7 animals.

### CFP procedure

The same behavioural procedure as in experiment 1 was used for training and for acquisition sessions of a *flavour-taste* CFP.

### FAL procedure

Once the flavour preference test (Test 2) had been completed, the rats were allowed a period of 48 h with food and water *ad libitum*. After 2 days with water available for 15 min/day from two tubes, all animals were offered 0.15% saccharin solution flavoured with 0.05% orange flavour. Half of the animals in each group) immediately received an intraperitoneal injection of either isotonic NaCl (0.9%) or LiCl (0.15 M, 20 ml/kg). The next day, a saccharin solution flavoured with a different flavour (tropical punch) was offered, in association with saline to the animals that had received LiCl the previous day and in association with LiCl to those that had received saline. Aversion was tested on day 5 in a choice test with two graduated cylinders containing flavours diluted in saccharin, considering the difference in consumption between them as the aversion index. In this FAL procedure, the flavours were always diluted in saccharin (acquisition sessions and Test) in order to minimize neophobia towards them and ensure that the animals would consume the largest amount of liquid possible before the LiCl administration.

### Histological procedures

After the behavioural testing, rats were anesthetized with sodium pentobarbital (80 mg/kg) and perfused intracardially with PBS, followed by 4% formaldehyde. Brains were removed and stored in formaldehyde. The relevant block of tissue was dehydrated through graded alcohols, embedded in paraffin, and sectioned at 5 μ. Sections were stained with hematoxylin and eosin to verify the lesion.

### Statistical analyses

Values were expressed as means ± SEM, and statistical analyses were performed using SPSS Statistics 20. A three-way repeated-measures ANOVA (group × trials × fluid) was used to analyze the mean intakes of flavours during the four acquisition trials and preference tests. Two-way repeated-measures ANOVA (group × fluid) was used to analyze the mean intakes in the aversion test, and two-way repeated-measures ANOVA (group × day) to analyze the effects of treatment on the body weight. Separate one-way repeated-measures ANOVA evaluated mean intakes during the two-bottle tests (CS+ *vs*. CS−) in lesioned and sham-lesioned groups. When a significant *F* was obtained, Newman-Keuls *post hoc* tests were performed to assess specific comparisons. Statistical significance was determined at the 5% level.

### Histology

**Figure 8:**
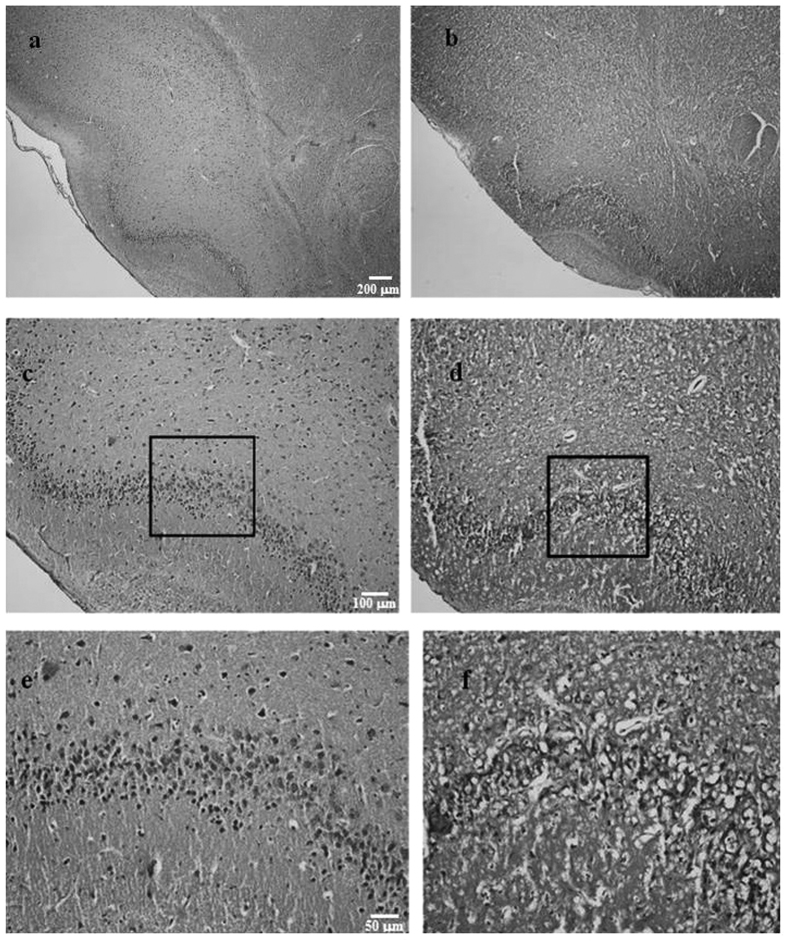
Representative photomicrographs (4x, 10x, 20x) depicting neurotoxic lesions in pyramidal cell layer 2 of the aPC (**b,d,f**) and intact structures in a sham-lesioned animal (**a,c,e**).

The extent of histological lesions was determined by the lack of somata in pyramidal cell layer 2 of the aPC, which were almost completely absent in some cases (see Fig. 8). Rostrocaudally, the lesions were limited to a small region between 3.72 and 2.16 anterior to the bregma^36^. Quantitative analysis of a representative section (−2.28 anterior a bregma), performed using ImageJ image analysis software, revealed a reduction of at least 50% of cells in this region in the lesioned animals in comparison to the controls.

## Additional Information

**How to cite this article**: Mediavilla, C. *et al*. Role of anterior piriform cortex in the acquisition of conditioned flavor preference. *Sci. Rep*. **6**, 33365; doi: 10.1038/srep33365 (2016).

## References

[b1] Berthoud H. Metabolic and hedonic drives in the neural control of appetite: who is the boss? Curr. Opinion Neurobiol. 21, 888–896 (2011). 10.1016/j.conb.2011.09.004 PMC3254791 21981809

[b2] Cooper S. J. Pharmacology of food, taste, and learned flavour preferences in Appetite and body weight. Integration system and the development of antiobesity drugs (eds Kirkhan, T. C. & Cooper, S. J.) 217–245 (Academic Press, 2007).

[b3] Houpt T. A., Philopena J. M., Wessel T. C., Joh T. H. & Smith G. P. Increased *c-fos* expression in nucleus of the solitary tract correlated with conditioned taste aversion to sucrose in rats. Neurosci. Letters 172, 1–5 (1994). 10.1016/0304-3940(94)90648-3 8084508

[b4] Swank M. W. & Bernstein I. L. c-Fos induction in response to a conditioned stimulus after single trial taste aversion learning. Brain Res. 636, 202–208 (1994). 8012803 10.1016/0006-8993(94)91018-9

[b5] Yamamoto T., Sacko N., Sakai N. & Iwafune A. Gustatory and visceral inputs to the amygdala of the rat: conditioned taste aversion and induction of c-*fos*-like immunoreactivity. Neurosci. Letters 226, 127–130 (1997). 10.1016/s0304-3940(97)00265-6 9159506

[b6] Gibson E. L. & Brunstrom J. M. Learned influences on appetite, food choice, and intake: Evidence in Human Beings in *Appetite and body weight*. Integration system and the development of antiobesity drugs (eds Kirkhan T. C. & Cooper S. J.) 271–300 (Academic Press, 2007).

[b7] Prescott J. Chemosensory learning and flavour: Perception, preference and intake. Physiol. Behav. 107, 553–559 (2012). 22525491 10.1016/j.physbeh.2012.04.008

[b8] Malkusz D. C. . Dopamine signaling in the medial prefrontal cortex and amygdala is required for the acquisition of fructose-conditioned flavor preferences in rats. Behav. Brain Res. 233, 500–507 (2012). 22579970 10.1016/j.bbr.2012.05.004 PMC3741658

[b9] Touzani K., Bodnar R. J. & Sclafani A. Activation of dopamine D1-like receptors in nucleus accumbens is critical for the acquisition, but not the expression, of nutrient-conditioned flavor preferences in rats. Eur. J. Neurosci. 27, 1525–1533 (2008). 18336564 10.1111/j.1460-9568.2008.06127.x

[b10] Uematsu A., Tsurugizawa T., Uneyama H. & Torii K. Brain-gut communication via vagus nerve modulates conditioned flavour preference. Eur J Neurosci. 31, 1136–1143 (2010). 20377626 10.1111/j.1460-9568.2010.07136.x

[b11] Haberly L. B. Parallel-distributed processing in olfactory cortex: new insights from morphological and physiological analysis of neuronal circuitry. Chem. Senses 26, 551–576 (2001). 11418502 10.1093/chemse/26.5.551

[b12] Maier J. X., Wachowiak M. & Katz D. B. Chemosensory convergence in the Primary Olfactory Cortex. J. Neurosci. 32, 17037–17047 (2012). 23197697 10.1523/JNEUROSCI.3540-12.2012 PMC3525522

[b13] Small D. M., Veldhuizen M. G. & Green B. Sensory Neuroscience: Taste responses in primary olfactory cortex. Curr. Biol. 23, 157–158 (2012). 10.1016/j.cub.2012.12.036 23428327

[b14] Illig K. R. Projections from orbitofrontal cortex to anterior piriform cortex in rat suggest a role in olfactory information processing. J. Comp. Neurol. 488, 224–231 (2005). 15924345 10.1002/cne.20595 PMC1360190

[b15] Calu D. J., Roesch M. R., Stalnaker T. A. & Schoenbaum G. Associative encoding in Posterior Piriform Cortex during odor discrimination and reversal learning. Cereb. Cortex 17, 1342–1349 (2007). 16882682 10.1093/cercor/bhl045 PMC2473864

[b16] Roesch M. R., Stalnaker T. A. & Schoenbaum G. Associative encoding in anterior piriform cortex versus orbitofrontal cortex during odor discrimination and reversal learning. Cereb. Cortex 17, 643–652 (2007). 16699083 10.1093/cercor/bhk009 PMC2396586

[b17] Jezzini A., Mazzucato L., La Camera G. & Fontanini A. Processing of hedonic and chemosensory features of taste in medial prefrontal and insular networks. J. Neurosci. 33, 18966–18978 (2013). 24285901 10.1523/JNEUROSCI.2974-13.2013 PMC3841457

[b18] Parabucki A. & Netser S. Origin of palatibility coding in medial prefrontal cortex. J. Neurosci. 34, 4121–4122 (2014). 24647933 10.1523/JNEUROSCI.0362-14.2014 PMC6608092

[b19] Taha S. A. & Fields H. L. Encoding of palatability and appetitive behaviors by distinct neuronal populations in the nucleus accumbens. J. Neurosci. 25, 1193–1202 (2005). 15689556 10.1523/JNEUROSCI.3975-04.2005 PMC6725953

[b20] Agustin-Pavon C., Parkinson J., Man M. & Roberts A. C. Contribution of the amygdala, but not orbitofrontal or medial prefrontal cortices, to the expression of flavour preferences in marmoset monkeys. Eur. J. Neurosci. 34, 1006–1017 (2011). 21848920 10.1111/j.1460-9568.2011.07813.x

[b21] Roullet F., Datiche F., Liénard F. & Cattarelli M. Learning-stage dependent Fos expression in the rat brain during acquisition of an olfactory discrimination task. Behav. Brain Res. 157, 127–137 (2005). 15617779 10.1016/j.bbr.2004.06.017

[b22] Gilbert P. E., Campbell A. & Kesner R. P. The role of the amygdala in conditioned flavor preference. Neurobiol. Learn. Mem. 79, 118–121 (2003). 12482686 10.1016/s1074-7427(02)00013-8

[b23] Risco S. & Mediavilla C. Orexin 1 receptor antagonist in central nucleus of the amygdala attenuates the acquisition of flavor-taste preference in rats. Pharmacol. Biochem. Behav. 126, 7–12 (2014). 25223979 10.1016/j.pbb.2014.09.002

[b24] Kelley A. E., Baldo B. A., Pratt W. E. & Will M. J. Corticostriatal-hypothalamic circuitry and food motivation: Integration of energy, action and reward. Physiol. Behav. 86, 773–795 (2005). 16289609 10.1016/j.physbeh.2005.08.066

[b25] Volman S. F. . New insights into the specificity and plasticity of reward and aversion encoding in the mesolimbic system. J. Neurosci. 33, 17569–17576 (2013). 24198347 10.1523/JNEUROSCI.3250-13.2013 PMC3818538

[b26] Capaldi E. D. Conditioned food preferences in Why we eat what we eat. The psychology of eating (ed. Capaldi E. D.) 53–80 (American Psychological Association, 2004).

[b27] Sclafani A. Psychobiology of food preferences. Int. J. Obesity 25, 13–16 (2001). 10.1038/sj.ijo.0801905 11840208

[b28] Cohen Y., Reuveni I., Barkai E. & Maroun M. Olfactory learning-induced long-lasting enhancement of descending and ascending synaptic transmission to the piriform cortex. J. Neurosci. 28, 6664–6669 (2008). 18579740 10.1523/JNEUROSCI.0178-08.2008 PMC6670420

[b29] Schoenbaum G. & Eichenbaum H. Information coding in the rodent prefrontal cortex. I. Single-neuron activity in orbitofrontal cortex compared with that in piryform cortex. J. Neurophysiol. 74, 733–750 (1995). 7472378 10.1152/jn.1995.74.2.733

[b30] Ueji K. & Yamamoto T. Flavor learningin weanling rats and its retention. Physiol. Behav. 106, 417–422 (2012). 22387575 10.1016/j.physbeh.2012.02.021

[b31] Scott T. R. Learning through the taste system. Front. Syst. Neurosci. 5, 1–6 (2011). 22131967 10.3389/fnsys.2011.00087 PMC3222881

[b32] Small D. M. & Prescott J. Odor/taste integration and the perception of flavor. Exp. Brain Res. 166, 345–357 (2005). 16028032 10.1007/s00221-005-2376-9

[b33] Small D. M. Flavor is in the brain. Physiol. Behav. 107, 540–552 (2012). 22542991 10.1016/j.physbeh.2012.04.011

[b34] Mediavilla C., Bernal A. & Puerto A. Taste aversion learning induced *c-fos* expression in the nucleus of the solitary tract after spontaneous flavor intake: Role of the inter-stimulus interval. Neurobiol. Learn. Mem. 88, 264–268 (2007). 17638581 10.1016/j.nlm.2007.05.008

[b35] Thiele T. E., Cubero I., van Dijk G., Mediavilla C. & Bernstein I. L. Ethanol-induced c-Fos expression in catecholamine-and Neuropeptide Y-producing neurons in rat brainstem. Alcohol Clin. Exp. Res. 24, 802–809 (2000). 10888068

[b36] Paxinos G. & Watson C. The rat brain in stereotaxic coordinates 5th ed. Academic Press, San Diego CA (2005).

